# Whether Cholera Is Contagious

**Published:** 1866-04

**Authors:** Jacob Bigelow


*Whether Cholera is Contagious.*—By JACOB BIGELOW, M. D.

Within the present century, cholera, a disease indigenous in hot climates of the East, has, at various intervals, made its appearance in the temperate latitudes of Europe and America. It is now again exciting interest from its possible and perhaps probable approach to this country.

The experience of the last thirty or forty years has led a majority of medical men who have observed the disease to believe that, as a general law, it is not contagious. In this belief I must individually remain, until evidence more satisfactory than any which has yet appeared shall justify an opposite conviction.

The great epidemic of 1830 and 1847 had a remarkable coincidence in the path which they pursued, and in the order and dates of their arrival in different cities. They seem to have followed certain great routes of travel, and to have avoided others equally frequented. According to Lesegue, they both visited consecutively, and in corresponding months, Tiflis, Astrachan, Moscow, Petersburg and Berlin. In 1831, cholera did not take the most frequented route from Berlin to Paris, but passed along the shores of the Baltic, crossed over to Sunderland, went down to London, and again crossed the channel and arrived in Paris about six months after its appearance at Berlin. A disease propagated by contagion of any kind would hardly have avoided the most frequented thoroughfares from Berlin to Paris, while it occupied half a year in going round by England.

The epidemic now or lately prevailing in Europe appears to date back at least nine months, at which time it existed among the caravans of pilgrims visiting or returning from the city of Mecca. In the middle of May last it was at Alexandria and Cairo, in June at Constantinople, Ancona and Marseilles, and in November at Paris, Havre, and other European cities.

Thus it appears that cholera has now existed in Europe from three to eight months, among cities having constant commercial intercourse with seaports of the United States, during which time thousands of passengers and tens of thousands of bales and packages have been landed in our maritime cities. If cholera were as contagious or portable as many believe it to be, it ought to have begun and perhaps finished its work in many of our seaports before this time.

Epidemics require two things for their introduction and extension. These are—first, predisposition in the inhabitants of the place visited; and, second, the arrival or presence of an exciting cause. This cause in some epidemics, such as small-pox, is contagion. In others it is an occult influence, not yet discovered nor understood, nor known to be controlled, except in some instances, by hygienic agencies. No country, I believe, has succeeded in keeping out cholera by quarantines, and no country, as far as we know, can produce it artificially or retain it after the predisposition has disappeared. In its own time it moves on thoroughfares where men are travelling, and spreads in cities where they are stationary, for no better known reason than that mankind are its necessary food, and that where there are no people there can be no cholera. But why, of two frequented roads or cities, it selects one and avoids the other, investigators have not yet been able to satisfy us.

The credit of having introduced the present epidemic into Europe, is by a sort of popular acclamation assigned to the hosts of squalid devotees who perform an annual pilgrimage to Mecca. Yet we are told that "the cholera exists every year among the caravans of Musselmans arriving at the holy cities," so that their supposed mission of forwarding the cholera to Europe, in most years fails to be performed.

Cholera, like influenza and some other migratory diseases, has usually but not always advanced from east to west. Of the vehicle in which it travels, or the course it is next to take, we know about as much as mankind knew of the cause of lightning before the discovery of electricity. Its conveyance and propagation have been ascribed to air, to water, to material foci, to electricity, to ozone or to the want of it. Of late, in consequence of the vast development by the microscope of the existence everywhere of minute living organisms, it has become more common to ascribe the arrival of this and other like epidemics to certain unseen "germs" which are called seeds or ova, cryptogamic or animalcular, according as the fancy of the theorist inclines him to adopt a vegetable or an animal nomenclature.

But in this, as in many other cases, it is easier to trace an analogy, or assume a cause, than it is to prevent an effect. Although inquirers have been indefatigable in their attempts to enlighten the world on the means of ridding ourselves of the various offensive cotenants of our globe, yet no crusade has yet succeeded in banishing from our fields and houses the unwelcome swarms of mosquitoes, worms, grubs and flies, which molest us with their annual presence; nor in suppressing the blight of grain, the potato rot, or the peachtree disease. Happily some, if not most of these have their periods of abatement or disappearance, and this rather through the order of Providence than the agency of man. Cholera seems to abide in the same category. We know little of its exciting cause, and not much of its prevention, except that by following in our personal habits the dictates of reason and experience, we diminish both the frequency and danger of its occurrence.

Whatever may be the cause or vehicle of cholera, credulous and excitable persons are impatient of suspense, and are prone to cut a knot which they fail to unite. When an epidemic disease first appears, some coincidence is always brought to light which is supposed capable of accounting for it. The arrival of a ship, the opening of a trunk or the washing of a garment, are among the most frequently accepted causes. But as these events have happened a thousand times before, and apparently under like circumstances, without any known results, it has been thought necessary by some of our later writers to narrow the compass of exposure down to the reception of the morbid exertions of individual into the digestive canal of another. The first impression made by this announcement must, if true, be one of relief, the danger not seeming likely to happen very often. But to the possibility of such danger we can never oppose an absolute negative, so long as we persist in eating smelts and flounders caught about the mouth of our drains, or even turnips, salads and strawberries raised at Brighton. The risk, however, is so small, that most persons will prefer to take it, rather than to deprive themselves of food or luxuries.

Of the many sensation tales printed and reprinted about cholera, and the supposed instanees of remarkable communication or arrestation, it is sufficient to say that they are frequently interesting, being fully as dramatic as they are probable.

In the same regard we cannot help noticing that credulity, and perhaps private cupidity, has caused much stress to be laid on the supposed preventive efficacy of what are called "disinfectants," a mysterious word which implies a thing assumed but not proved to exist. We have deodorizers, such as chlorine, charcoal, &c., which by their combinations render certain effluvia imperceptible to our sense. But that these are not *disinfectants*, there is most abundant evidence. The narrative, then, of the physician of Malta, who covered certain surfaces in vessels with oil, and had them "disinfected by chlorine gas," after which "no new cases occurred," is to be classed with other like results, with which the medical press always abounds at the close of epidemics.

In clean and well-regulated cities of temperate climates, cholera is far from being the most formidable of epidemics. A greater part of its victims are the miserably poor, the worn out, the ill provided, and the intemperate, in whom this disease only anticipates the date, but does not greatly increase the annual or biennial number of deaths. Its mortality in our northern Atlantic cities rarely amounts to one per cent. of the population in a given place or year, so that a man may reside through an epidemic in one of these cities with less risk than he can take a pleasure voyage to Europe. After having witnessed many cases of cholera in this and other cities, I am farther satisfied that it affords one of the easiest modes of exit from the world.

People who would avoid or prevent cholera should cultivate equanimity, regularity of life and habits, cleanliness, salubrious exercise, temperance, and avoidance of all excess. When they have done their duty in providing for the care of the sick, allaying public panics, and abating public nuisances, they may safely dismiss their apprehensions. Little good and some harm is always done by the indiscreet agitation of a subject which is to a great extent beyond our control. A single or sporadic case of cholera occurring in a village of a thousand inhabitants may attract little notice, and perhaps pass without record; but a hundred cases in a city of a hundred thousand inhabitants make an aggregate which generally causes some panic, though the proportion is exactly the same, and the panic equally unnecessary. It is possible that the supposed immunity of country districts in comparrison with cities may be accounted for by the fact, that in the sparce population of country towns cases are less liable to be detected and published.

I may be excused for repeating the following remarks from among some "Aphorisms" published by me about thirty years ago, when the disease was new and little known among us. "Should cholera contine to prevail for three years throughout this continent, it would cease to interrupt either business or recreation. Mankind cannot always stand aghast, and the wheels of society at length would be no more impeded by its presence than they now are by the existence of consumption, old age or drunkenness."

